# Net Clinical Benefit of Left Atrial Appendage Occlusion After Intracerebral Hemorrhage in Atrial Fibrillation: A Focused Review and Benchmark-Adjusted Secondary Analysis

**DOI:** 10.3390/jcm15218612

**Published:** 2026-11-05

**Authors:** Christos Kourek, Serge Sicouri, Alexandros Briasoulis, Dimitrios E. Magouliotis, Andrew Xanthopoulos, Ioannis Paraskevaidis, Basel Ramlawi

**Affiliations:** 1 Department of Cardiac Surgery Research, Lankenau Institute for Medical Research, Wynnewood, PA 19096, USA; sicouris@mlhs.org (S.S.); dmagouliotis@gmail.com (D.E.M.); ramlawib@mlhs.org (B.R.); 2 Amyloidosis Center of Excellence, Department of Therapeutics, National and Kapodistrian University of Athens, 11528 Athens, Greece; alexbriasoulis@gmail.com; 3 Department of Cardiology, University Hospital of Larissa, 41334 Larissa, Greece; andrewvxanth@gmail.com; 4 Medical School of Athens, National and Kapodistrian University of Athens, 11527 Athens, Greece; iparas@otenet.gr; 5 Department of Cardiac Surgery, Lankenau Medical Center, Wynnewood, PA 19096, USA

**Keywords:** atrial fibrillation, intracerebral hemorrhage, left atrial appendage occlusion, net clinical benefit, stroke, bleeding, decision analysis

## Abstract

**Background/Objectives**: Atrial fibrillation (AF) after intracerebral hemorrhage (ICH) creates a therapeutic conflict: withholding oral anticoagulation may increase ischemic stroke risk, whereas resumption of anticoagulation may increase recurrent ICH. Left atrial appendage occlusion (LAAO) offers a nonpharmacologic alternative, but isolated stroke or bleeding rates do not describe the overall benefit–harm balance. This focused review examines net clinical benefit (NCB) as a decision framework for LAAO after ICH and presents a secondary benchmark-adjusted analysis derived from our prior systemaatic review and meta-analysis. **Methods**: Thirteen endocardial LAAO cohorts with complete data for ischemic stroke, recurrent ICH, major bleeding, and all-cause death contributed to the analysis. The component event data and prespecified severity weights were carried forward from the prior meta-analysis; the external benchmark comparison, study-level benchmark-adjusted NCB synthesis, and benchmark-sensitivity analyses were not part of that report and constitute the distinct secondary analysis presented here. **Results**: Prespecified external benchmark rates were 8.0, 2.0, 3.0, and 6.0 events per 100 patient-years, respectively. Unweighted and severity-weighted NCB were evaluated using both pooled-rate and study-level Hartung–Knapp random-effects approaches. Study-level pooled NCB was 12.20 events per 100 patient-years (95% CI, 8.61–15.79; I^2^ = 96%), and weighted NCB was 14.39 (95% CI, 8.84–19.94; I^2^ = 94%). Additional two-component analyses using PRESTIGE-AF reference rates yielded unweighted NCB estimates of 5.65 and 2.06 events per 100 patient-years for no-OAC and DOAC comparator scenarios, respectively. Excluding all-cause mortality from the original model yielded an unweighted NCB of 8.41. These exploratory results highlight the sensitivity of NCB estimates to comparator selection and endpoint composition. **Conclusions**: Contemporary randomized evidence, including PRESTIGE-AF, CLOSURE-AF, and CHAMPION-AF, reinforces that benefit–harm balance is population-, comparator-, endpoint-, and time-horizon-dependent. Accordingly, these NCB estimates should be interpreted as decision-analytic and hypothesis-generating rather than causal treatment effects. ICH-specific randomized comparisons remain essential.

## 1. Introduction

Stroke prevention in patients with atrial fibrillation (AF) who have survived intracerebral hemorrhage (ICH) remains a major therapeutic challenge because cardioembolic protection must be balanced against the risk of recurrent cerebral bleeding. Our recent systematic review and meta-analysis found relatively low observed rates of ischemic stroke and recurrent ICH after left atrial appendage occlusion (LAAO), but the predominantly observational evidence does not by itself establish the overall benefit–harm balance of the procedure [1]. Net clinical benefit (NCB) provides a framework for integrating competing outcomes rather than considering stroke and bleeding separately. This concept was established in AF anticoagulation by weighting thromboembolic prevention against intracranial hemorrhage according to their differing clinical consequences [2].

The background risk in AF survivors of ICH is substantial [3]. Randomized studies further illustrate the therapeutic tradeoff. SoSTART and APACHE-AF demonstrated high vascular-event rates and uncertainty regarding the optimal strategy after intracranial hemorrhage [4,5]. The COCROACH individual-participant meta-analysis suggested that restarting oral anticoagulation reduces ischemic major cardiovascular events, while its effect on hemorrhagic events remains uncertain [6]. More recently, PRESTIGE-AF showed a marked reduction in ischemic stroke with direct oral anticoagulation compared with no anticoagulation, but recurrent ICH was more frequent [7]. These findings demonstrate why assessment of a single endpoint may provide an incomplete picture of treatment value.

LAAO offers a nonpharmacologic strategy that may reduce thromboembolic risk without requiring indefinite systemic anticoagulation. A propensity-matched study suggested more favorable composite outcomes with LAAO than with standard medical care after ICH, although residual confounding remains unavoidable [8]. Contemporary registry data support the feasibility of LAAO in patients with prior intracranial hemorrhage [9], while a recent meta-analysis confirmed the growing observational evidence base in patients with previous intracranial hemorrhage or cerebral amyloid angiopathy (CAA) [10]. The LAA-CAA cohort further demonstrated the feasibility of LAA closure in patients with severe CAA and AF [11]. Consistent with these data, contemporary US and European AF guidelines recognize percutaneous LAAO as an option for selected patients in whom long-term anticoagulation is contraindicated or carries an unacceptable bleeding risk [12,13].

Accordingly, the present focused review examines NCB as a decision framework for LAAO after ICH and presents a secondary benchmark-adjusted analysis integrating ischemic stroke, recurrent ICH, major bleeding, and mortality using unweighted and severity-weighted approaches.

## 2. Why Net Clinical Benefit Is Especially Relevant After ICH

### 2.1. Competing Hazards Are Not Symmetric

The post-ICH AF population sits at the intersection of two different disease mechanisms. Cardioembolic stroke is driven principally by atrial cardiomyopathy, blood stasis, and thrombus formation within the left atrial appendage, whereas recurrent spontaneous ICH often reflects small-vessel pathology such as hypertensive arteriopathy or CAA. Consequently, reducing one hazard does not necessarily reduce the other. Anticoagulation can markedly lower ischemic stroke risk while simultaneously increasing the probability or severity of recurrent bleeding. Conversely, avoiding anticoagulation protects against drug-mediated hemorrhagic risk but leaves the cardioembolic substrate untreated. The Danish nationwide cohort used to inform the present benchmark framework illustrates the high background burden in ICH survivors with AF and the importance of considering ischemic events, recurrent hemorrhage, and mortality together [3].

Randomized data emphasize the same tension. SoSTART and APACHE-AF were underpowered for definitive comparative conclusions but established the feasibility of randomizing ICH survivors and demonstrated substantial vascular event rates regardless of strategy [4,5]. In the COCROACH individual-participant meta-analysis, starting OAC reduced ischemic major adverse cardiovascular events, while the effect on hemorrhagic major adverse cardiovascular events remained uncertain [6]. PRESTIGE-AF then provided the clearest randomized demonstration of the tradeoff: DOAC therapy substantially reduced ischemic stroke compared with no anticoagulation, but recurrent ICH was more frequent and the prespecified noninferiority criterion for recurrent ICH was not met [7]. These findings make a single-outcome interpretation clinically incomplete.

### 2.2. Conventional Scores Do Not Solve the Post-ICH Decision

CHA_2_DS_2_-VASc and related thromboembolic scores estimate embolic risk but were not designed to integrate the mechanism, location, or imaging phenotype of a prior ICH. Likewise, bleeding scores are best viewed as tools to identify modifiable bleeding risk factors rather than instruments for withholding anticoagulation. The 2024 European Society of Cardiology (ESC) AF guideline explicitly notes the modest predictive performance of bleeding scores and highlights situations such as ICH related to CAA in which OAC may need to be avoided [13]. The 2023 ACC/AHA/ACCP/HRS AF guideline similarly lists spontaneous intracranial or intraspinal bleeding due to a nonreversible cause among situations in which long-term anticoagulation is contraindicated and considers percutaneous LAAO reasonable in patients with sufficient stroke risk and such a contraindication [12].

NCB therefore should not be interpreted as a replacement for clinical judgment or risk stratification. Its role is complementary: to make explicit the assumptions clinicians already make implicitly about the frequency and severity of competing outcomes, and to expose how the preferred strategy changes when those assumptions change.

## 3. Contemporary Treatment Landscape: Why Comparator Choice Matters

### 3.1. OAC Resumption After ICH

The comparator against which LAAO is judged has changed materially over the past decade. “No anticoagulation” is not a homogeneous strategy: some patients receive antiplatelet therapy, some receive no antithrombotic therapy, and many undergo delayed reassessment after blood-pressure optimization and neurologic characterization. In parallel, DOACs have replaced vitamin K antagonists for most eligible patients. COCROACH suggested a reduction in ischemic events with OAC after intracranial hemorrhage without a statistically definitive estimate for hemorrhagic harm [6]. PRESTIGE-AF sharpened the tradeoff by reporting markedly fewer ischemic strokes but more recurrent ICH with DOAC therapy versus no anticoagulation [7]. Thus, an NCB estimate that uses a “medical management” benchmark must state whether it represents untreated natural history, antiplatelet-treated patients, contemporary DOAC use, or a mixture of these approaches.

### 3.2. LAAO Evidence Outside the Prior-ICH Population

Randomized LAAO trials provide essential context but cannot simply be transplanted to ICH survivors. PRAGUE-17 showed noninferiority of LAAO to DOAC therapy for a broad composite in high-risk patients, with long-term follow-up suggesting less nonprocedural bleeding after LAAO [14,15]. In 2026, two large randomized trials further changed the evidence landscape. CLOSURE-AF enrolled patients at high risk of both stroke and bleeding and did not establish noninferiority of LAAO to physician-directed best medical care for a composite that included stroke, systemic embolism, major bleeding, and cardiovascular or unexplained death [16]. By contrast, CHAMPION-AF enrolled anticoagulation-eligible patients and found LAAO noninferior to non-vitamin K antagonist oral anticoagulation for cardiovascular death, stroke, or systemic embolism, while non-procedure-related bleeding was lower with LAAO [17].

These apparently divergent results are not contradictory when viewed through an NCB lens. Procedural risk is front-loaded, bleeding benefit accrues over time, comparator therapy differs, baseline risk differs, and composite definitions determine which events dominate an analysis. A 2026 meta-analysis of randomized LAAO-versus-DOAC trials similarly found reduced non-procedure-related bleeding with LAAO but a possible tradeoff in ischemic stroke depending on trial selection [18]. For patients with prior ICH, the relevant balance may differ substantially because recurrent intracranial bleeding has a higher baseline probability and greater clinical weight than in anticoagulation-eligible populations.

## 4. Focused Review Methods and Benchmark-Adjusted NCB Framework

This focused review integrates contemporary randomized trials, guidelines, consensus documents, and key observational studies relevant to AF after ICH and LAAO. The quantitative source dataset derives from our previously published systematic review and meta-analysis [1], but the present manuscript does not repeat its pooled clinical endpoint, neuroimaging, timing, antithrombotic, or epicardial analyses. Thirteen endocardial LAAO cohorts with complete data for ischemic stroke, recurrent ICH, major bleeding, and all-cause death were used solely for the secondary NCB analysis.

The present analysis represents a distinct secondary investigation based on aggregate data previously extracted for our systematic review and meta-analysis [1]. The earlier publication focused on observed clinical event rates following LAAO and explored the influence of hemorrhagic phenotype, procedural timing, and postprocedural antithrombotic therapy. In contrast, the present analysis examines the sensitivity of a benchmark-adjusted NCB framework to external comparator selection, endpoint composition, severity weighting, and underlying statistical assumptions. No new patients or clinical observations were added.

The comprehensive search strategy, eligibility criteria, study-selection procedures, and assessment of potentially overlapping cohorts are detailed in our original systematic review and meta-analysis, currently in press [1]. The present focused review incorporates contemporary randomized evidence for narrative context, while its secondary quantitative analysis is restricted to 13 cohorts from the original dataset with complete outcome information.

Benchmark-adjusted net clinical benefit (NCB) was defined as the difference between expected comparator event rates and observed LAAO event rates, expressed per 100 patient-years. The original exploratory reference scenario incorporated ischemic stroke, recurrent ICH, major bleeding, and all-cause mortality, with expected rates of 8.0, 2.0, 3.0, and 6.0 events per 100 patient-years, respectively (Table 1). These rates were selected as pragmatic assumptions informed by published natural-history and medical-management studies [3,6,7] rather than derived from a single homogeneous comparator population. Accordingly, this reference scenario represents a hypothetical decision-analytic model rather than a direct treatment comparison.

**Table 1:** Base-case inputs to the benchmark-adjusted net clinical benefit framework.

Component	Expected Rate Per 100 PY	Severity Weight	Role in NCB
Ischemic stroke	8.0	1.0	Thromboembolic efficacy
Recurrent ICH	2.0	1.5	Catastrophic intracranial bleeding risk
Major bleeding	3.0	0.7	Clinically important extracranial bleeding
All-cause death	6.0	2.0	Competing mortality burden

ICH indicates intracerebral hemorrhage; NCB, net clinical benefit; PY, patient-years. Expected comparator rates are external benchmarks and are not concurrent randomized control rates. The 1.5 weighting for intracranial hemorrhage is consistent with the classic AF net-clinical-benefit framework [2]. Weights assigned to major bleeding and all-cause death were prespecified decision-analytic assumptions and should not be interpreted as validated patient utilities .

The four reference rates were prespecified modeling assumptions and should not be interpreted as directly observed event rates from a single medical-management cohort. Source-specific estimates illustrate the variability between populations and treatment strategies. For example, PRESTIGE-AF reported ischemic stroke rates of 8.60 (95% CI, 5.43–12.80) and 0.83 (95% CI, 0.14–2.57) per 100 patient-years in the no-OAC and DOAC groups, respectively, with corresponding recurrent ICH rates of 0.82 (95% CI, 0.14–2.53) and 5.00 (95% CI, 2.68–8.39) [7]. The alternative comparator scenarios and probabilistic sensitivity analyses were therefore used to explore the dependence of NCB on benchmark selection and uncertainty rather than to establish a definitive comparative effect.

To assess the influence of comparator selection, additional analyses were conducted using treatment-arm event rates reported in the randomized PRESTIGE-AF trial [7]. Separate scenarios were constructed for patients receiving no oral anticoagulation (OAC) and those receiving direct oral anticoagulants (DOACs). Ischemic stroke rates were 8.60 and 0.83 events per 100 patient-years, while recurrent ICH rates were 0.82 and 5.00 events per 100 patient-years, respectively. Because compatible treatment-arm rates for major bleeding and all-cause mortality could not be consistently established, these analyses were restricted to ischemic stroke and recurrent ICH and were evaluated separately from the original four-component model.

For each cohort, unweighted NCB was calculated as the sum of expected minus observed event rates, while severity-weighted NCB incorporated prespecified weights of 1.0 for ischemic stroke, 1.5 for recurrent ICH, 0.7 for major bleeding, and 2.0 for all-cause mortality. Mathematically, NCB was calculated as Σ_k_ w_k_(B_k_ − O_k_), where B_k_ and O_k_ represent the external benchmark and observed LAAO event rates for each outcome, respectively, and w_k_ represents its assigned severity weight (w_k_ = 1 for all components in the unweighted model). Positive values indicate a lower observed aggregate event burden relative to the assumed benchmark, rather than a causal reduction in clinical events. All-cause mortality was included as an adverse component in this arithmetic framework, but competing-risk relationships could not be formally modeled using the available aggregate data. Unweighted NCB represents the net difference between expected and observed aggregate adverse-event rates, whereas severity-weighted NCB represents the corresponding difference after applying prespecified relative severity weights. Neither measure should be interpreted as the number of unique patients experiencing or avoiding an adverse event. Cohort-specific estimates were synthesized using inverse-variance random-effects models with DerSimonian–Laird estimation of between-study variance and Hartung–Knapp adjustment of confidence intervals. Within-study variances were approximated using Poisson event counts, with continuity corrections for zero-event components when required. External comparator rates were treated as fixed within each scenario. Additional sensitivity analyses varied the original benchmark rates independently and simultaneously by ±25%, excluded all-cause mortality to examine potential overlap between fatal and nonfatal events, excluded the large NCDR cohort reported by Mansour et al. to assess its influence, and evaluated cohorts explicitly classified as spontaneous-ICH-only in the extraction dataset. The latter analysis was considered exploratory because hemorrhage subtype could not be consistently established across all contributing cohorts. All analyses used previously extracted aggregate data without additional patient-level observations.

To further assess the dependence of NCB on model assumptions, we performed a break-even analysis to identify the proportional reduction in all external benchmark rates required for NCB to reach zero. We also conducted an exploratory probabilistic sensitivity analysis using 250,000 Monte Carlo iterations per scenario. External benchmark rates were modeled using independent lognormal distributions with coefficients of variation of 25% and 50%, representing alternative hypothetical levels of uncertainty. Severity weights were either held at their original values or varied using triangular distributions for recurrent ICH (1.0–1.5–2.0), major bleeding (0.3–0.7–1.0), and all-cause mortality (1.0–2.0–3.0), while ischemic stroke remained the reference outcome with a weight of 1.0. The observed pooled LAAO component rates were held fixed during these simulations. Additional deterministic analyses examined alternative severity-weight combinations. These analyses were designed to characterize the influence of assumed benchmark and weight uncertainty rather than establish comparative treatment effectiveness.

Study-level variances were initially estimated using Poisson approximations based on the observed event counts and total person-time, with continuity corrections applied to zero-event components. Because the NCB score has a fixed upper bound equal to the expected benchmark burden, conventional normal-approximation confidence intervals may extend beyond its mathematically permissible range, particularly in small cohorts with no recorded events. We therefore examined exact Poisson-based intervals for the unweighted zero-event observations and performed a sensitivity analysis excluding cohorts with zero recorded events across all components. We additionally calculated approximate 95% prediction intervals from the random-effects models to illustrate the dispersion of effects expected across populations. These conventional prediction intervals were interpreted cautiously because the normal-scale model does not enforce the theoretical upper bound of NCB. Leave-one-out sensitivity analyses were additionally performed by sequentially excluding each contributing cohort and repeating the random-effects synthesis. Meta-regression was not undertaken because of the limited number of eligible cohorts and incomplete reporting of relevant study-level covariates, which would have limited the reliability and clinical interpretation of such analyses.

The primary analysis incorporated all-cause mortality as an adverse outcome alongside ischemic stroke, recurrent ICH, and major bleeding. However, death was also recognized as a terminal competing event, and fatal ischemic or hemorrhagic events could contribute to more than one component. Because the analysis relied on aggregate published data, mutually exclusive fatal and nonfatal events could not be consistently identified. A sensitivity analysis excluding all-cause mortality was therefore performed to assess the influence of this component on the estimated NCB.

The time origin for observed LAAO outcomes was generally the intervention or subsequent follow-up, whereas the external benchmark studies could use earlier time origins, including the index hemorrhagic event. Consequently, these populations were not aligned at a common treatment-decision time point, introducing potential survivor-selection and immortal-time-related bias. Periprocedural complications were not modeled as a separate NCB component. Events occurring during the procedural period could contribute to the relevant clinical endpoints when included in the original study definitions and extracted outcome counts; however, their ascertainment and reporting were not sufficiently consistent to permit a separate quantitative analysis.

The severity weights were selected to reflect differences in the clinical consequences of the evaluated outcomes. Ischemic stroke was assigned a reference weight of 1.0, while recurrent ICH received a weight of 1.5, consistent with the established NCB framework proposed by Singer et al. [2]. Major bleeding was assigned a lower weight of 0.7 to reflect its generally lower severity compared with intracranial hemorrhage, whereas all-cause mortality received the highest weight of 2.0 because of its irreversible consequences. Importantly, the weights assigned to major bleeding and mortality represent prespecified decision-analytic assumptions rather than empirically validated patient-derived utilities. Fatal ischemic strokes or hemorrhagic events may contribute to both their respective clinical endpoints and all-cause mortality, potentially resulting in double counting. Because individual-patient data were unavailable, this overlap could not be quantified or eliminated. Furthermore, the substantial between-study heterogeneity may reflect differences in patient characteristics, hemorrhagic risk profiles, procedural techniques, follow-up duration, and outcome ascertainment. Therefore, the resulting NCB estimates should be interpreted as exploratory measures of adverse-event burden rather than validated patient-level utilities or causal treatment effects.

Potential overlap between published cohorts was considered when selecting the quantitative dataset. The analysis was restricted to 13 study cohorts with extractable data for all four NCB components. Where the same underlying population could have been represented in more than one publication, cohort independence was assessed using reported recruitment periods, participating centers, patient characteristics, and study definitions. Because individual-patient identifiers were unavailable, undetected overlap between independently published observational cohorts cannot be definitively excluded. For transparency and reproducibility, the cohort-level event counts and person-time used in the present calculations are provided in Appendix A Table A1.

## 5. NCB Findings

### 5.1. Study-Level NCB

**Figure 1:**
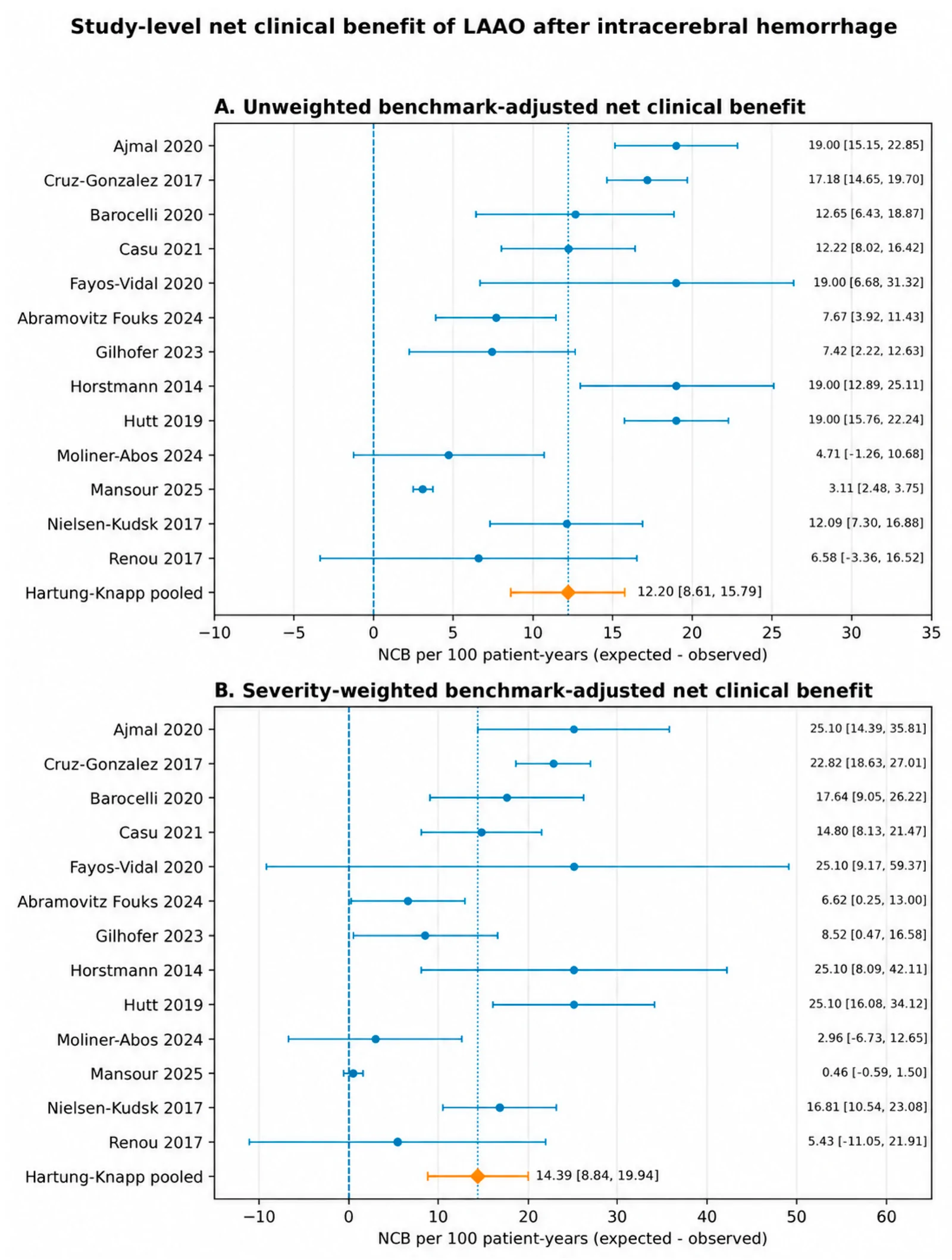
Study-level benchmark-adjusted net clinical benefit after LAAO in patients with prior intracerebral hemorrhage. Panel A shows unweighted NCB and Panel B shows severity-weighted NCB. Study-specific estimates are expressed per 100 patient-years as expected minus observed adverse-event burden. Positive values indicate lower observed burden relative to the prespecified external benchmark.

Across 13 contributing cohorts, study-level NCB estimates were generally positive but varied substantially (Figure 1). The Hartung–Knapp random-effects pooled unweighted NCB was 12.20 events per 100 patient-years (95% CI, 8.61–15.79), with very high heterogeneity (I^2^ = 96%). The corresponding severity-weighted NCB was 14.39 per 100 patient-years (95% CI, 8.84–19.94; I^2^ = 94%). The high I^2^ values are not a peripheral statistical detail; they indicate that the magnitude of benchmark-adjusted benefit differs markedly across cohorts and should not be interpreted as a stable treatment effect applicable to an individual patient.

Four smaller cohorts (Ajmal et al., Fayos-Vidal et al., Horstmann et al., and Hutt et al.) had zero recorded events across all four NCB components, producing the maximum possible unweighted and severity-weighted scores of 19.00 and 25.10 per 100 patient-years, respectively. However, their limited sample sizes and follow-up, together with potential differences in outcome ascertainment, raise concerns regarding the precision and interpretation of these estimates. Excluding these four cohorts reduced the pooled unweighted NCB to 9.38 (95% CI, 5.82–12.94; I^2^ = 94.6%) and the severity-weighted NCB to 10.88 (95% CI, 4.91–16.84; I^2^ = 94.8%). The reduction in the pooled estimates indicates that the zero-event cohorts influenced the overall results, although substantial heterogeneity persisted.

The approximate 95% prediction intervals were −6.19 to 30.59 for the unweighted model and −12.08 to 40.86 for the severity-weighted model. Both included zero, indicating considerable uncertainty regarding the expected NCB in a new population. Because these conventional normal-scale intervals extend beyond the theoretical maximum NCB values, they should be interpreted as indicators of substantial between-study dispersion rather than as fully bounded predictive estimates. The sensitivity analysis excluding zero-event cohorts and the corresponding prediction intervals are summarized in Appendix A Table A2.

In leave-one-out sensitivity analyses, pooled unweighted NCB estimates ranged from 11.57 to 13.17 per 100 patient-years, while severity-weighted estimates ranged from 13.39 to 15.69. Exclusion of any individual cohort did not reverse the direction of the pooled point estimates. However, substantial heterogeneity persisted, with I^2^ ranging from 79.8% to 96.3% for unweighted analyses and from 71.4% to 94.5% for weighted analyses. These findings indicate that the pooled estimates were not driven exclusively by a single cohort, but do not establish their applicability to individual patients or different ICH populations (Appendix A Table A3).

### 5.2. Pooled-Rate NCB

The complementary pooled-rate analysis yielded an unweighted NCB of 10.23 (95% CI, 6.29–12.67) and a severity-weighted NCB of 12.16 (95% CI, 5.98–16.00) per 100 patient-years. These intervals were obtained by combining the marginal 95% confidence limits of the observed pooled LAAO component rates while treating the external benchmark rates and severity weights as fixed.

### 5.3. Sensitivity to Benchmark Assumptions

**Figure 2:**
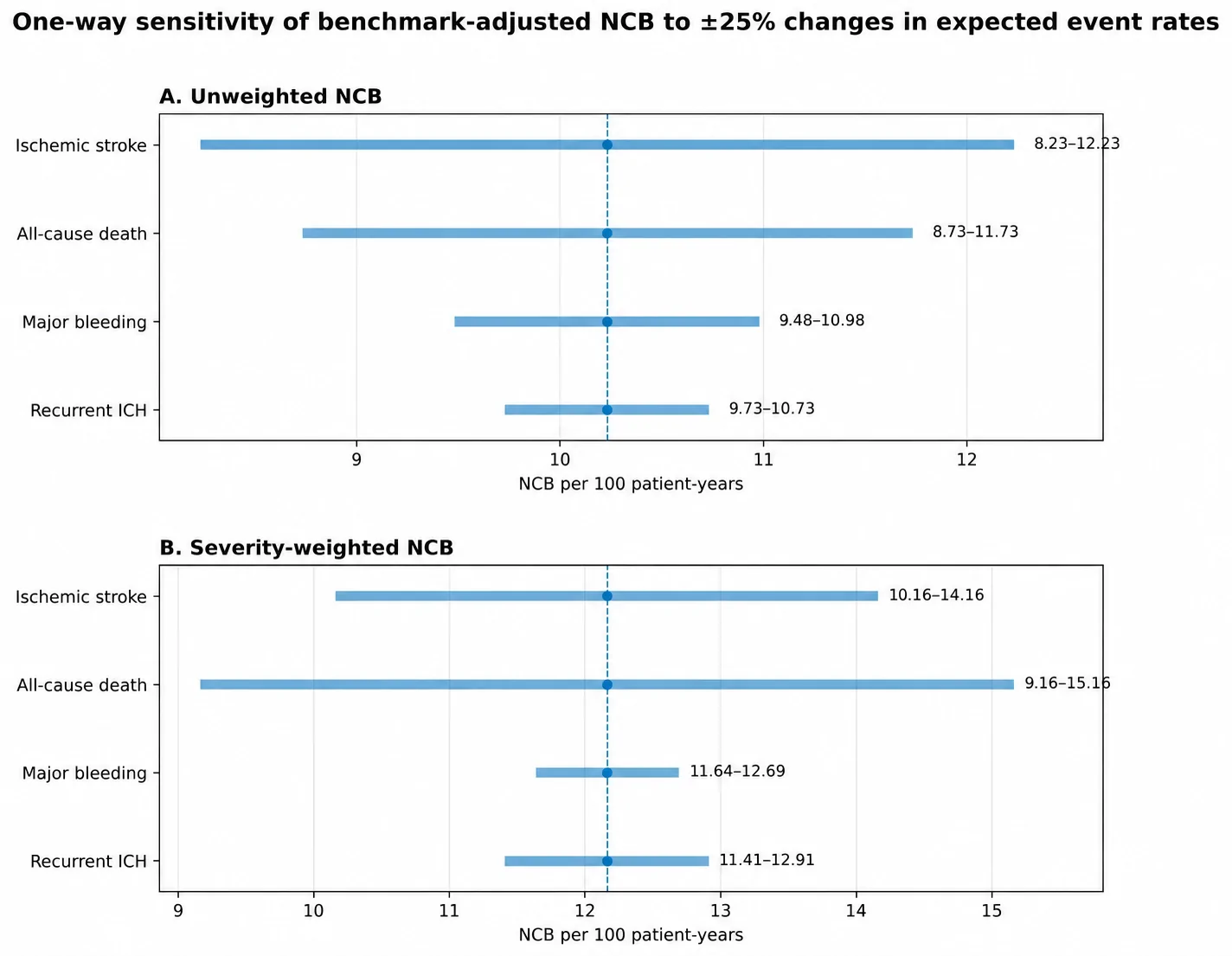
Sensitivity of benchmark-adjusted net clinical benefit to comparator assumptions. One-way sensitivity analysis varies each expected comparator rate by ±25% while holding all other inputs fixed. Panel A shows unweighted NCB and Panel B shows severity-weighted NCB.

In one-way sensitivity analyses, unweighted NCB remained positive when each external benchmark was varied independently by ±25% (Figure 2). The largest shifts occurred when the expected ischemic stroke or all-cause mortality rate was changed. With the severity-weighted formulation, mortality assumptions exerted the largest influence because death carried a weight of 2.0, followed by ischemic stroke. The direction of NCB was unchanged under each one-way scenario.

A post hoc simultaneous sensitivity scenario produced the same qualitative conclusion while illustrating the importance of absolute baseline risk (Table 2). If all four expected rates were reduced by 25%, unweighted NCB decreased from 10.23 to 5.48 per 100 patient-years and weighted NCB decreased from 12.16 to 5.89 per 100 patient-years. If all expected rates were increased by 25%, the corresponding NCB estimates increased to 14.98 and 18.44 per 100 patient-years. These deterministic calculations illustrate the mathematical sensitivity of NCB to proportional changes in the assumed benchmark rates. Because the resulting changes are directly determined by the magnitude of each benchmark and its assigned severity weight, preservation of positive NCB across the examined scenarios should not be interpreted as independent evidence of robustness. The analyses were therefore complemented by break-even, probabilistic, and severity-weight sensitivity assessments.

### 5.4. Comparator-Specific NCB Scenarios

In exploratory analyses restricted to ischemic stroke and recurrent ICH, the magnitude of benchmark-adjusted NCB varied according to the selected medical-management comparator. Using the no-OAC event rates from PRESTIGE-AF [7], the pooled unweighted NCB was 5.65 events per 100 patient-years (95% CI, 5.33–5.98), while the severity-weighted estimate was 5.20 (95% CI, 4.79–5.60). When DOAC-treated patients were used as the external reference, the corresponding estimates were 2.06 (95% CI, 1.74–2.39) and 3.70 (95% CI, 3.29–4.10), respectively.

These results demonstrate the sensitivity of the estimated NCB to the comparator strategy and to the relative weighting of ischemic and hemorrhagic outcomes. Importantly, these two-component analyses are not directly comparable in magnitude with the original four-component model, which also incorporated major bleeding and all-cause mortality. Furthermore, the confidence intervals reflect uncertainty in the observed LAAO data under fixed external comparator rates and do not incorporate uncertainty in the benchmark estimates or differences in baseline risk between populations. Therefore, these results should not be interpreted as evidence of comparative treatment superiority.

These findings demonstrate the sensitivity of the estimated NCB to the selected comparator strategy, with lower unweighted estimates when DOAC-treated patients were used as the external reference (Appendix A Table A4).

### 5.5. Additional Sensitivity Analyses

Excluding all-cause mortality from the original benchmark-adjusted model yielded an unweighted NCB of 8.41 events per 100 patient-years (95% CI, 6.89–9.94) and a severity-weighted NCB of 8.05 (95% CI, 6.57–9.54). Compared with the original four-component estimates, these results demonstrate the substantial influence of mortality on the model. Although the point estimates remained positive, this analysis does not eliminate potential overlap between recurrent ICH and major bleeding or account for differences in time origin and patient selection between the LAAO cohorts and external comparator populations (Appendix A Table A4).

Exclusion of the large NCDR cohort reported by Mansour et al. resulted in an unweighted NCB of 13.17 (95% CI, 9.76–16.59; I^2^ = 79.8%) and a weighted NCB of 15.69 (95% CI, 10.54–20.84; I^2^ = 71.4%). Although heterogeneity decreased, substantial between-study variation persisted.

A restricted analysis of the two cohorts explicitly identified as spontaneous-ICH-only in the available extraction dataset yielded an unweighted four-component NCB of 5.21 (95% CI, −5.24 to 15.66) and a weighted NCB of 3.59 (95% CI, −10.14 to 17.33). These estimates were imprecise and should be interpreted cautiously, given the small number of eligible cohorts and incomplete reporting of hemorrhage subtype in other studies. The findings therefore do not establish the NCB of LAAO specifically in patients with spontaneous ICH.

### 5.6. Break-Even and Probabilistic Sensitivity Analyses

In the pooled-rate framework, the estimated NCB reached zero when all four external benchmark rates were reduced simultaneously by approximately 53.8% in the unweighted model and 48.4% in the severity-weighted model. These thresholds represent algebraic properties of the assumed reference rates and observed LAAO event burdens rather than independently validated clinical thresholds.

In exploratory probabilistic sensitivity analyses incorporating uncertainty in the benchmark rates and severity weights, the weighted NCB remained positive in nearly all simulations under the examined assumptions. With benchmark coefficients of variation of 25% and varying severity weights, the median NCB was 11.72 per 100 patient-years (95% simulation interval, 5.69–20.92). Increasing benchmark uncertainty to a coefficient of variation of 50% reduced the median NCB to 10.85 (95% simulation interval, 1.01–30.49), with approximately 98.8% of simulations yielding positive values. However, these findings were conditional on the prespecified probability distributions and fixed observed LAAO rates and did not incorporate uncertainty arising from cohort selection, between-population differences, or correlated clinical outcomes.

Additional deterministic sensitivity analyses demonstrated that the magnitude of NCB varied across alternative severity-weight assumptions (Appendix A Table A5). These analyses differed from the benchmark-rate sensitivity analyses presented in Table 2 and Figure 2, in which severity weights remained fixed. The findings emphasize that the estimated NCB depends on the relative clinical importance assigned to individual outcomes and should not be interpreted as evidence of causal treatment superiority.

The results of the probabilistic sensitivity analyses under different benchmark uncertainty assumptions and severity-weight distributions are presented in Appendix A Table A6. Additional severity-weight sensitivity analyses demonstrated variation in NCB magnitude across alternative weighting assumptions (Appendix A Table A5).

## 6. How Should a Positive NCB Be Interpreted Clinically?

### 6.1. NCB Is a Decision Model, Not a Randomized Treatment Effect

The central interpretive point is that “positive NCB” does not mean that LAAO causally prevents 10 to 14 adverse events per 100 patient-years. The benchmarks are external, nonconcurrent, and assembled from different populations and treatment eras. Selection for LAAO is highly nonrandom: patients who survive long enough to undergo the procedure, are accepted by a multidisciplinary team, and can tolerate peri-procedural antithrombotic therapy differ from patients managed medically. Residual differences in frailty, ICH mechanism, neuroimaging phenotype, blood-pressure control, renal function, adherence, and competing mortality can all shift observed rates independently of the procedure.

The only direct propensity-matched comparison in the literature on prior ICH, by Nielsen-Kudsk and colleagues, reported a lower composite rate of ischemic stroke, major bleeding, or all-cause mortality with LAAO than with standard medical care [8]. That study is clinically supportive but cannot eliminate residual confounding. The large NCDR LAAO Registry analysis further demonstrates that patients with prior ICH remain a distinct high-risk subgroup even after LAAO, reinforcing the need to avoid extrapolating results from general LAAO populations [9].

The influence of sparse data is particularly relevant to the interpretation of the study-level synthesis. Four small cohorts had no recorded events across the evaluated outcomes, resulting in maximum possible NCB estimates. Although removing these cohorts reduced the pooled estimates, substantial heterogeneity remained, and the approximate prediction intervals included zero. In contrast, the large contemporary NCDR cohort yielded a substantially smaller weighted NCB estimate, with a confidence interval crossing zero. These findings illustrate the sensitivity of the pooled results to study characteristics, event ascertainment, and statistical weighting. They also highlight that a positive pooled NCB estimate should not be interpreted as evidence of consistent benefit across all patient populations.

The additional comparator-specific analyses further illustrate the importance of selecting a clinically coherent reference population. The estimated NCB was lower when DOAC-treated patients were considered instead of patients receiving no oral anticoagulation, reflecting the substantially different balance between ischemic stroke prevention and recurrent intracerebral hemorrhage observed in contemporary randomized evidence. Nevertheless, these findings should not be interpreted as demonstrating the superiority of LAAO over either treatment strategy, since the compared populations were not randomized against each other and differed in clinical characteristics and treatment eligibility. In addition, the two-component estimates are conditional on external event-rate assumptions and may change when other outcomes, including major bleeding and mortality, are incorporated. The proposed framework is therefore most appropriately used to explore how competing clinical risks influence decision-making rather than to provide a definitive ranking of therapeutic strategies.

### 6.2. Weighting Matters Because Events Are Not Clinically Interchangeable

An unweighted composite implicitly assumes that a nonfatal major extracranial bleed, a disabling ischemic stroke, recurrent lobar ICH, and death carry equivalent clinical importance. They do not. Severity weighting is therefore conceptually appropriate, but the weights are value judgments. The classic AF NCB framework assigned intracranial hemorrhage 1.5 times the impact of thromboembolism based on observed mortality and disability [2]. Our expanded framework also includes major bleeding and all-cause death, but those weights have not been prospectively validated in an ICH-specific population. Different patients may rationally assign different utilities: an independent older adult with probable CAA may place extraordinary disutility on recurrent ICH, whereas another patient may prioritize avoidance of disabling ischemic stroke or may view a procedure-related complication differently from late spontaneous bleeding.

For this reason, future NCB work should move beyond fixed expert-selected weights toward patient-derived utilities, quality-adjusted life-years, or disability-weighted endpoints. At minimum, published NCB analyses should report unweighted results, weighted results, and sensitivity analyses across plausible weight ranges rather than presenting a single number as definitive.

### 6.3. Time Horizon Changes the Balance

LAAO has an early procedural hazard and a potential late benefit from avoiding chronic anticoagulant exposure. An annualized event-rate framework can blur this temporal asymmetry. CLOSURE-AF is instructive because early procedure-related events contributed to the composite and affected the trial-level benefit–harm balance (Table 3) [16]. Conversely, CHAMPION-AF demonstrated a lower burden of non-procedure-related bleeding over three years in anticoagulation-eligible patients [17]. In an ICH survivor, the clinically relevant time horizon may depend on life expectancy, frailty, recurrence risk, and the expected duration of postimplant antithrombotic therapy. A two-year NCB and a five-year NCB need not have the same sign or magnitude.

**Table 3:** Evidence that should inform an ICH-specific NCB discussion.

Evidence Source	Population	Comparison	Main Relevance to NCB	Prior Intracranial-Hemorrhage Population
SoSTART [4]	203 ICrH survivors with AF	Start vs. avoid OAC	Feasibility; uncertain recurrent-ICH tradeoff	Yes
APACHE-AF [5]	101 anticoagulation-associated ICH survivors	Apixaban vs. avoid OAC	High vascular event burden under both strategies	Yes
COCROACH [6]	412 randomized participants pooled	Start vs. avoid OAC	Lower ischemic events; hemorrhagic effect uncertain	Yes
PRESTIGE-AF [7]	319 spontaneous ICH survivors	DOAC vs. no OAC	Strong ischemic benefit offset by more recurrent ICH	Yes
Nielsen-Kudsk et al. [8]	Matched AF + prior ICH cohorts	LAAO vs. standard care	Only direct matched LAAO comparison; residual confounding	Yes
CLOSURE-AF [16]	High stroke + bleeding risk AF	LAAO vs. best medical care	Shows importance of procedural harm and comparator quality	No; contextual
CHAMPION-AF [17]	Anticoagulation-eligible AF	LAAO vs. NOAC	Noninferior efficacy; less nonprocedural bleeding	No; contextual
PRAGUE-17 [14,15]	High-risk AF	LAAO vs. DOAC	Long-term composite and bleeding context	No; contextual

AF, atrial fibrillation; ICH, intracerebral hemorrhage; ICrH, intracranial hemorrhage; LAAO, left atrial appendage occlusion; NCB, net clinical benefit; NOAC/DOAC, non-vitamin K antagonist/direct oral anticoagulant; OAC, oral anticoagulation.

Intracardiac echocardiography (ICE) has emerged as an alternative to transesophageal echocardiography for procedural guidance during LAAO. In a retrospective analysis of 411 patients undergoing ICE-guided LAAO, Pastormerlo et al. reported a procedural success rate of 94.9% and major adverse events in 5.1% of patients, predominantly pericardial tamponade (2.9%). Despite favorable procedural outcomes, all-cause mortality reached 45.3% over a mean follow-up of 44.2 months, highlighting the substantial competing mortality burden in this population [19]. These findings emphasize that procedural success alone cannot establish long-term net clinical benefit. However, the retrospective, single-center design and absence of an ICH-specific analysis limit direct extrapolation to patients with previous spontaneous ICH.

Future NCB analyses should distinguish early procedural events (≤30 days), intermediate outcomes (31 days–1 year), and longer-term outcomes (>1 year) to better characterize the temporal balance between procedural hazards and potential benefits. Ideally, procedure-related complications should be incorporated from a common treatment-decision time point, with mutually exclusive endpoint definitions where appropriate. Individual-patient data would also permit competing-risk, multistate, and recurrent-event analyses that account for correlated outcomes and the terminal nature of death. Such analyses could not be reliably performed in the present study because of inconsistent procedural-event reporting and the absence of individual-patient event times.

### 6.4. Recurrent Events and Competing Death Require Better Methods

The present event-burden framework permits more than one event from the same patient and differs from a conventional time-to-first-event composite. That feature can be an advantage when recurrent bleeding or recurrent ischemia materially affects quality of life, but it also complicates interpretation because events within a patient are correlated. All-cause death is additionally a terminal competing event that prevents subsequent stroke or bleeding. Future analyses should therefore consider recurrent-event models, multistate models, competing-risk estimands, and restricted mean event-free time. The 2026 Left Atrial Appendage Academic Research Consortium recommendations on standardized endpoint definitions provide an important foundation for making such comparisons more coherent across trials [20].

An additional methodological consideration is the potential overlap between fatal and nonfatal clinical events. For example, a fatal ischemic stroke or recurrent ICH may be recorded under both the respective clinical endpoint and all-cause mortality. This may increase the apparent adverse-event burden, particularly when mortality receives a higher severity weight. Since the analysis was based on aggregate published data, mutually exclusive fatal and nonfatal events could not be consistently distinguished across cohorts. Future patient-level analyses should account for this overlap through hierarchical composite endpoints, competing-risk approaches, or appropriately defined multistate models.

A further concern is the difference in time origin between the LAAO cohorts and the external comparator populations. Patients undergoing LAAO generally enter follow-up at the time of intervention, whereas natural-history cohorts may begin observation at the index hemorrhagic event. Since patients must survive and remain eligible until LAAO, early recurrent hemorrhagic events and deaths occurring before implantation are not captured in the LAAO follow-up period. This survivor-selection mechanism may favor the intervention in indirect comparisons and cannot be adequately corrected using aggregate event rates. In addition, procedural complications were not evaluated as an independent NCB component, although some may have been captured within the reported stroke, bleeding, or mortality outcomes. These limitations reinforce the need for future comparisons that align treatment strategies at a common decision time point, include early procedural harms, and distinguish fatal from nonfatal events.

## 7. Translating NCB into a Practical Post-ICH Decision Framework

An NCB-oriented clinical discussion should start with patient selection rather than with the device. The first step is to characterize the index hemorrhage: spontaneous versus traumatic, lobar versus deep, probable CAA versus hypertensive arteriopathy, and the presence of microbleeds or cortical superficial siderosis. The second step is to estimate the untreated or minimally treated thromboembolic burden, considering age, prior ischemic stroke, vascular disease, heart failure, AF pattern, and left atrial substrate. The third step is to determine whether the contraindication to long-term OAC is permanent, potentially reversible, or uncertain. The fourth step is to assess LAAO-specific risk, including vascular access, pericardial complications, device-related thrombus, residual leak, and the ability to tolerate the necessary short-term postprocedural antithrombotic regimen.

This approach is consistent with contemporary guidelines and practical consensus statements, which emphasize individualized assessment and shared decision-making rather than a universal “LAAO versus OAC” rule [12,13,21]. The relevant question for a patient with prior ICH is not whether LAAO is globally superior to DOAC therapy in AF. It is whether, for that patient’s hemorrhagic mechanism, thromboembolic risk, procedural risk, life expectancy, and preferences, the expected long-term event burden of LAAO is lower than the plausible alternatives.

From a practical perspective, the proposed NCB framework may support individualized decision-making by integrating the patient’s thromboembolic risk, hemorrhagic recurrence risk, procedural risk, and expected survival. Patients with lobar ICH, probable cerebral amyloid angiopathy, multiple cerebral microbleeds, or cortical superficial siderosis may have particularly high risks of recurrent intracranial bleeding, potentially favoring consideration of LAAO when long-term anticoagulation is contraindicated. Conversely, patients with deep hypertensive ICH, well-controlled blood pressure, and an acceptable bleeding risk may remain candidates for anticoagulation following multidisciplinary assessment. Additional factors, including frailty, renal function, life expectancy, anticipated postprocedural antithrombotic therapy, and patient preferences, should inform the final treatment strategy. The proposed framework is therefore intended to facilitate structured shared decision-making rather than establish a universal threshold for selecting LAAO over medical therapy.

## 8. What the Recent Randomized Trials Add to the NCB Question

The most recent trials make two lessons especially relevant to an ICH-focused NCB framework. First, NCB is population-specific. CHAMPION-AF enrolled patients who were suitable candidates for anticoagulation and therefore had a fundamentally different hemorrhagic-risk distribution from patients with prior spontaneous ICH [17]. Its favorable reduction in non-procedure-related bleeding after LAAO is encouraging but does not quantify the balance in patients with CAA, multiple lobar microbleeds, or a recent anticoagulation-associated ICH. Second, NCB is endpoint specific. CLOSURE-AF is instructive because early post-procedural strokes and major bleeding events contributed to the time-to-event composite, allowing the front-loaded procedural hazard of LAAO to influence the overall benefit–harm balance [16]. A framework that excludes early procedural complications can make a device strategy appear more favorable than a trial that counts them.

Berti et al. recently proposed a phenotype-based framework distinguishing CHAMPION-like patients, characterized by greater physiological reserve and sufficient life expectancy to realize longer-term benefits, from CLOSURE-like patients, in whom frailty, multimorbidity, procedural risk, and competing mortality may limit the potential benefit of LAAO [22]. This framework further emphasizes the importance of individualized assessment of hemorrhagic risk, renal function, postprocedural antithrombotic tolerance, and time to benefit. However, neither phenotype can be directly extrapolated to patients with previous spontaneous ICH, who require additional consideration of hemorrhage mechanism and recurrence risk.

These lessons argue for an ICH-specific NCB model that begins at the time of treatment allocation, includes procedural complications, distinguishes intracranial from extracranial bleeding, captures recurrent events, and uses a clinically meaningful follow-up horizon. They also argue against applying a single benchmark across all ICH survivors. Deep hypertensive ICH with excellent blood-pressure control, lobar ICH with probable CAA, traumatic ICH, and subdural hemorrhage represent biologically different recurrence risks and should not be treated as interchangeable states.

## 9. Limitations and Research Agenda

Several additional limitations should be acknowledged. First, the four-component benchmark was hypothetical and did not represent a homogeneous medical-management population. Alternative PRESTIGE-AF scenarios included only ischemic stroke and recurrent ICH, excluding major bleeding and mortality. The reported uncertainty intervals did not account for benchmark uncertainty or correlations between outcomes. Furthermore, fatal strokes or hemorrhages could overlap with all-cause mortality, while major bleeding definitions could include intracranial events. Although mortality-excluded analyses yielded positive NCB estimates, potential double counting could not be fully eliminated.

In addition, differences in hemorrhage definitions and patient characteristics limited comparability across cohorts. Some studies included broader intracranial hemorrhage populations, whereas others did not adequately specify ICH subtype. The spontaneous-ICH-only analysis included just two cohorts and yielded imprecise estimates. Substantial heterogeneity further limited the generalizability of pooled results. Moreover, probabilistic sensitivity analyses relied on hypothetical benchmark and severity-weight distributions without incorporating uncertainty or correlations in observed LAAO outcomes. Thus, simulation intervals and probabilities of positive NCB should not be interpreted as conventional confidence intervals or evidence of treatment superiority, while break-even thresholds remain mathematical rather than validated clinical thresholds.

Furthermore, all-cause mortality was influenced by frailty, comorbidities, and survival to implantation. Differences in time origin between LAAO and comparator cohorts introduced potential survivor-selection and immortal-time bias. Inconsistent reporting of periprocedural complications and the absence of individual-patient event times precluded reliable landmark, competing-risk, multistate, and recurrent-event analyses.

Finally, this secondary analysis shared its observational dataset with our previous meta-analysis [1] and should not be considered independent clinical evidence. Potential patient overlap could not be definitively excluded, while differences in outcome reporting, reconstructed person-time, device characteristics, and postprocedural antithrombotic regimens introduced additional uncertainty. Collectively, these limitations prevent causal interpretation and underscore the need for patient-level studies with standardized endpoints, aligned treatment-decision time points, and directly comparable populations.

## 10. Conclusions

For patients with AF who have survived ICH, the central therapeutic problem is not the isolated risk of ischemic stroke or recurrent bleeding but the balance between both, together with major bleeding, procedural complications, and mortality. A benchmark-adjusted NCB framework makes that balance explicit. In the present secondary analysis of 13 LAAO cohorts, NCB was positive across study-level, pooled-rate, unweighted, weighted, and benchmark-sensitivity analyses, but the magnitude varied substantially and depended on external assumptions. Although the benchmark-adjusted NCB estimates were generally positive, their magnitude was sensitive to comparator selection, endpoint composition, and the inclusion of small zero-event cohorts. The substantial between-study heterogeneity, prediction intervals encompassing zero, and considerably smaller estimates in the large contemporary NCDR cohort limit the generalizability of the pooled findings. Accordingly, the proposed framework should be regarded as an exploratory decision-analytic approach rather than evidence of uniform or causal clinical benefit from LAAO. Further studies using consistent endpoint definitions, appropriate count-based statistical methods, and directly comparable treatment populations are necessary.

## Figures and Tables

**Table 2:** Sensitivity of net clinical benefit to ±25% changes in expected benchmark rates.

Scenario	Expected-Rate Change	Unweighted NCB	Weighted NCB
Base case	None	10.23	12.16
Ischemic stroke	−25% to +25%	8.23–12.23	10.16–14.16
Recurrent ICH	−25% to +25%	9.73–10.73	11.41–12.91
Major bleeding	−25% to +25%	9.48–10.98	11.64–12.69
All-cause death	−25% to +25%	8.73–11.73	9.16–15.16
All four rates simultaneously	−25%	5.48	5.89
All four rates simultaneously	+25%	14.98	18.44

ICH, intracerebral hemorrhage; NCB, net clinical benefit; PY, patient-years. Values represent benchmark-adjusted NCB per 100 patient-years. One-way sensitivity analyses varied each expected comparator event rate independently by ±25%, holding all other rates and severity weights constant. Simultaneous analyses varied all four expected rates by ±25%. Severity weights were fixed at 1.0 for ischemic stroke, 1.5 for recurrent ICH, 0.7 for major bleeding, and 2.0 for all-cause mortality. All analyses used the original pooled LAAO event-rate estimates and hypothetical external benchmarks rather than concurrent randomized comparator data.

## Data Availability

This secondary analysis was based on previously extracted aggregate data from published observational studies included in our systematic review and meta-analysis, currently in press in the Canadian Journal of Cardiology [1]. Additional analytical datasets are available from the corresponding author upon reasonable request.

## Abbreviations

The following abbreviations are used in this manuscript:

AF	Atrial fibrillation
CAA	Cerebral amyloid angiopathy
CI	Confidence interval
DOAC	Direct oral anticoagulant
ICH	Intracerebral hemorrhage
LAAO	Left atrial appendage occlusion
NCB	Net clinical benefit
OAC	Oral anticoagulation
PY	Patient-years

## Appendix A

**Table A1:** Cohort-level data, follow-up characteristics, and outcome events included in the benchmark-adjusted net clinical benefit analysis.

Study	Patients (*n*)	Person-Years	Follow-Up (Months)	Ischemic Stroke (*n*)	Recurrent ICH (*n*)	Major Bleeding (*n*)	All-Cause Death (*n*)
Ajmal 2020 [23]	16	36.00	27.0	0	0	0	0
Cruz-González 2017 [24]	47	109.67	28.0	1	1	0	0
Barocelli 2020 [25]	31	63.00	24.2	2	0	1	1
Casu 2021 [26]	110	147.58	16.1	2	3	1	4
Fayos-Vidal 2020 [27]	9	11.25	15.0	0	0	0	0
Abramovitz-Fouks 2025 [28]	146	309.03	25.4	6	6	3	20
Gilhofer 2023 [29]	134	164.10	14.7	2	2	6	9
Horstmann 2014 [30]	20	22.67	13.6	0	0	0	0
Hutt 2019 [31]	38	42.75	13.5	0	0	0	0
Moliner-Abós 2024 [32]	40	154.00	46.2	3	6	3	10
Mansour 2025 [9]	14,539	15,144.79	12.5	317	267	484	1338
Nielsen-Kudsk 2017 [8]	151	115.80	6.0	2	1	3	2
Renou 2017 [33]	46	48.30	12.6	2	1	0	3

**Table A2:** Sensitivity of study-level benchmark-adjusted NCB to exclusion of zero-event cohorts and between-study heterogeneity.

Analysis	Cohorts (*n*)	Pooled NCB (95% CI)	I^2^ (%)	Approximate 95% Prediction Interval
Unweighted—original model	13	12.20 (8.61–15.79)	96.0	−6.19 to 30.59
Unweighted—excluding zero-event cohorts	9	9.38 (5.82–12.94)	94.6	−7.20 to 25.95
Weighted—original model	13	14.39 (8.84–19.94)	94.0	−12.08 to 40.86
Weighted—excluding zero-event cohorts	9	10.88 (4.91–16.84)	94.8	−15.85 to 37.60

NCB, net clinical benefit; CI, confidence interval. NCB values are expressed per 100 patient-years. Four cohorts (Ajmal 2020, Fayos-Vidal 2020, Horstmann 2014, and Hutt 2019) with zero recorded events across all four components were excluded in sensitivity analyses. Estimates were obtained using random-effects meta-analysis with Hartung–Knapp confidence intervals. Prediction intervals were calculated using a conventional normal-scale approximation and may extend beyond the theoretical upper bound of NCB (19.0 unweighted and 25.1 weighted).

**Table A3:** Leave-one-out sensitivity analyses of study-level benchmark-adjusted net clinical benefit.

Measure	Unweighted NCB	Severity-Weighted NCB
Original 13-cohort estimate	12.20	14.39
Lowest leave-one-out estimate	11.57	13.39
Highest leave-one-out estimate	13.17	15.69
Range of I^2^ across exclusions	79.8–96.3%	71.4–94.5%

All NCB estimates are per 100 patient-years. Comparisons are exploratory and nonrandomized.

**Table A4:** Comparator-specific and additional sensitivity analyses of benchmark-adjusted net clinical benefit after LAAO.

Analysis	Unweighted NCB (95% CI)	Weighted NCB (95% CI)
Original reference, four outcomes	12.20 (8.61–15.79)	14.39 (8.84–19.94)
No OAC, stroke + recurrent ICH	5.65 (5.33–5.98)	5.20 (4.79–5.60)
DOAC, stroke + recurrent ICH	2.06 (1.74–2.39)	3.70 (3.29–4.10)
Original model excluding mortality	8.41 (6.89–9.94)	8.05 (6.57–9.54)
Original model excluding Mansour 2025	13.17 (9.76–16.59)	15.69 (10.54–20.84)
Spontaneous-ICH-only, original reference	5.21 (−5.24–15.66)	3.59 (−10.14–17.33)

All values are events per 100 patient-years. Comparisons are exploratory and nonrandomized. No-OAC and DOAC scenarios use only ischemic stroke and recurrent ICH; all other analyses use the original four outcomes unless specified.

**Table A5:** Severity-weight sensitivity analysis.

Weight Scenario	Stroke	ICH	Bleeding	Death	NCB
Original weights	1.0	1.5	0.7	2.0	12.16
Equal weights	1.0	1.0	1.0	1.0	10.23
Lower hemorrhage/death weights	1.0	1.0	0.3	1.0	9.03
Higher hemorrhage/death weights	1.0	2.0	1.0	3.0	15.15
Excluding mortality	1.0	1.5	0.7	0.0	7.52

These are pooled-rate arithmetic scenarios.

**Table A6:** Probabilistic sensitivity analysis.

Benchmark CV	Weight Assumptions	Median Weighted NCB	95% Simulation Interval	Probability NCB > 0
25%	Fixed	11.87	5.80 to 20.33	≈100%
25%	Varying	11.72	5.69 to 20.92	≈100%
50%	Fixed	11.01	1.10 to 29.90	98.8%
50%	Varying	10.85	1.01 to 30.49	98.8%

All NCB values are per 100 patient-years. These are Monte Carlo simulation intervals, not confidence intervals for clinical treatment effects.
